# No one is immune to misinformation: An investigation of misinformation sharing by subscribers to a fact-checking newsletter

**DOI:** 10.1371/journal.pone.0255702

**Published:** 2021-08-10

**Authors:** Lauren L. Saling, Devi Mallal, Falk Scholer, Russell Skelton, Damiano Spina

**Affiliations:** 1 School of Health & Biomedical Science, RMIT University, Melbourne, Victoria, Australia; 2 FactLab RMIT University, Melbourne, Victoria, Australia; 3 RMIT ABC Fact Check, Melbourne, Victoria, Australia; 4 School of Computing Technologies, RMIT University, Melbourne, Victoria, Australia; University of Haifa, ISRAEL

## Abstract

Like other disease outbreaks, the COVID-19 pandemic has led to the rapid generation and dissemination of misinformation and fake news. We investigated whether subscribers to a fact checking newsletter (n = 1397) were willing to share possible misinformation, and whether predictors of possible misinformation sharing are the same as for general samples. We also investigated predictors of willingness to have a COVID-19 vaccine and found that although vaccine acceptance was high on average, it decreased as a function of lower belief in science and higher conspiracy mentality. We found that 24% of participants had shared possible misinformation and that this was predicted by a lower belief in science. Like general samples, our participants were typically motivated to share possible misinformation due to interest in the information, or to seek a second opinion about claim veracity. However, even if information is shared in good faith and not for the purpose of deceiving or misleading others, the spread of misinformation is nevertheless highly problematic. Exposure to misinformation engenders faulty beliefs in others and undermines efforts to curtail the spread of COVID-19 by reducing adherence to social distancing measures and increasing vaccine hesitancy.

## 1. Introduction

The COVID-19 pandemic has produced a massive demand for information concerning, for instance, the origin of the virus, routes of transmission, prevention, disease severity, and disease management. It has also generated misinformation, often grounded in conspiracy theories, which are readily believed in spite of the fact that such claims may be implausible and are not verified as accurate. The World Health Organization (WHO) has referred to the problem of large amounts of misinformation spread during the COVID-19 pandemic as an “infodemic” [1]. Fake news is rapidly disseminated online [2] and social media is a primary outlet for the spread of fake news and misinformation, given that these platforms are widely used for news consumption in general. Misinformation has also been found to generate more interest and emotional engagement than real news because of its novelty [3]. For example, fake news disseminated during the 2016 US presidential election accrued far more attention in the form of likes, shares and reactions on Facebook than real news [4]. The generation of fake news and its capacity to gain traction has increased in tandem with the rise of social media platforms as popular sources of news and information acquisition.

A mistrust of scientific information and belief in the accuracy of misinformation substantially undermines efforts to manage the pandemic and limit the spread of COVID-19 [2] For instance, preventative measures such as mask wearing and physical distancing are only effective if they are adhered to by the majority of individuals, but such measures are less likely to be followed if the scientific information underpinning them is not trusted. Similarly, misinformation undermines vaccine acceptance [5]. Misinformation is insidious as once false information forms the basis for a false belief, it is very hard to change the belief [6].

Whether an individual is likely to believe misinformation and fake news is determined both by individual characteristics and contextual factors. For instance, a lack of trust in science and low numeracy skills have been found to correlate with susceptibility to misinformation [7]. Individuals may also be susceptible to fake news because they trust the news source, or because it offers ideologically reinforcing information, that is, information that coheres with pre-existing beliefs [8]. For instance, conservatives may be more likely to uncritically accept claims made on Fox news, especially when such content confirms prior beliefs, but may argue vehemently against claims made on CNN in order to defend prior beliefs [9]. Although a behavioural heuristic such as this may be helpful to reduce the cognitive burden on an individual—because it means that one doesn’t have to evaluate each claim individually—the heuristic can result in the generation of false beliefs.

Pennycook and Rand [10] suggest that it is not faulty reasoning but instead a lack of reasoning that undermines the capacity to assess the accuracy of news. Similarly, Martel, Pennycook [11] demonstrated the crucial role of reasoning in distinguishing fake news from genuine news. The authors found that heightened emotionality as well as reliance on emotion rather than reasoning, increased belief in the accuracy of fake news. Getting people to reflect on the accuracy of claims increases their capacity to distinguish fake news from genuine news [12] because it makes claim veracity more salient [13]. The evidence suggests, therefore, that it is not a cavalier attitude to the truth that makes people susceptible to fake news, but rather a focus of attention on irrelevant factors that derails veracity judgements.

Although belief in misinformation is problematic, the spreading of misinformation and fake news is more problematic because it promotes faulty beliefs in others [14] and because exposure to misinformation on one occasion promotes belief in the veracity of that misinformation when it is encountered subsequently [15].

Social media platforms are particularly powerful outlets for the spread of fake news—special credence is given to information circulated on these platforms because it is often shared by people who are trusted and may be circulated in the context of closed groups [16]. Furthermore, unlike legacy media publishing outlets such as newspapers, radio, and television, social media platforms do not have regulated editorial standards, so misinformation can be published and widely circulated before it is identified and removed.

The inclination to *share* fake news and misinformation should be distinguished from the susceptibility to *believe* such information. This is because individuals who are susceptible to believing fake news may not be inclined to spread it while people who share misinformation may not judge it to be accurate [12]. However, the sharing of misinformation may, in some cases, provide a behavioural marker for a cognitive orientation to trust misinformation.

It is worth noting that the phrase ‘sharing misinformation’ is ambiguous between multiple phenomena, as follows: (i) the sharing of false information which you believe to be true; (ii) the sharing of false information which, for all you know, could be either true or false; and, (iii) the sharing of false information which you believe to be false. Previous studies have sometimes failed to distinguish phenomena (i) and (ii); however, we have attempted to distinguish them in the present study.

Willingness to share misinformation has been demonstrated to vary as a function of individual differences. For instance, older, politically conservative individuals have been found to be more willing to share misinformation than their younger more liberal counterparts [17]. Similarly, aspects of political belief including social dominance orientation, has been found to predict the sharing of misinformation [18]. It was thus of interest in the present study to determine whether predictors of the inclination to share misinformation (or possible misinformation) in our sample are comparable to predictors in general samples.

Motivations for sharing misinformation, or possible misinformation, can vary and do not necessarily involve a desire to mislead or trick others. Rather, there are many different motivations for sharing fake news and misinformation, including a desire to generate discussion about the veracity of the information and a desire to warn others about questionable content. And people are not motivated to share fake news as they report that it is important to share accurate news but lapses in attention to claim veracity promotes misinformation sharing [12].

Even without a motivation to mislead others, sharing information that you are not sure is true can lead others to develop faulty beliefs, so you end up participating in the spread of misinformation. Perhaps there is a kind of naivety associated with sharing misinformation even if one’s motivation for sharing is not problematic. Given that sharing false information occurs even when the information is judged to be inaccurate [12], our focus in this study was on the sharing of possible misinformation rather than on the susceptibility to belief in misinformation.

A prevailing concern in the context of COVID-19 is vaccine hesitancy which can be promoted by misinformation. Although a number of vaccines have been developed to prevent COVID-19 or its effects, there is substantial vaccine hesitancy in the population. For instance, one study found that 35% of Irish participants and 31% of participants from the UK were reluctant to have a vaccine [19]. A systematic review revealed vaccine hesitancy rates of around 30% in many countries [20]. A recent survey by Essential Research [21], which looked into the issue of vaccine hesitancy in Australia, found that the proportion of respondents who said “I’ll never get vaccinated” increased from around 10% at the start of March 2021 to around 16% in late April 2021. This change may be related to the potential risk of blood clots that were identified for the AstraZeneca vaccine. Moreover, the proportion of females who say they would never get vaccinated (20%) is substantially higher than for males (12%).

In order for vaccines to be effective in preventing the spread of COVID-19 and achieving herd immunity, between 70% and 80% of the population need to be vaccinated [22]. Therefore, investigating factors that influence vaccine hesitancy (including psychological and demographic factors) is of great importance and was investigated here.

### 1.1 The present study

Clearly there are a range of factors that influence the likelihood that an individual will sharing misinformation, including inattention to accuracy [7], the platform on which the claim is delivered [8], and psychological factors. Bringing the accuracy of information and sources to the forefront of attention seems to reduce the likelihood that false information will be shared [12]. Given this, one would predict that individuals who are i. concerned about fake news and misinformation, ii. clearly aware of the importance of judging claim accuracy, and iii. actively seek debunking of questionable claims, would not share possible misinformation. This was investigated in the present study by recruiting a subscribers of the CoronaCheck newsletter.

#### 1.1.1 About the CoronaCheck newsletter

The CoronaCheck newsletter was launched on 27 March 2020 by RMIT ABC Fact Check. RMIT ABC Fact Check is Australia’s premier fact-checking organisation, and one of approximately 105 fact-checking organisations worldwide accredited by the International Fact-Checking Network (IFCN). The CoronaCheck weekly newsletter publishes fact-checking of COVID-related news. As of March 2021, the newsletter had 16,000 subscribers; on average, 45% of recipients open the newsletter. The newsletter is also published on the ABC News website with an average weekly readership of 92,000.

#### 1.1.2 Aims

The present study focussed on individuals who have elected to receive regular information about possible fake news regarding COVID-19. The aim of the study was to investigate how these individuals engage with misinformation and to see how this compares with the more general population. Specifically, our study was guided by the following research questions:

1. Do CoronaCheck newsletter subscribers share possible misinformation?
2. What are the predictors of possible misinformation sharing in this cohort, and how does this compare with predictors of this behaviour in general samples?
3. How trustworthy do subscribers find expert scientific information about COVID-19 and its vaccines to be?
4. Does willingness to have the COVID-19 vaccine in this cohort vary as a function of individual differences?

## 2. Method

### 2.1 Participants

An a priori power analysis conducted using G*Power 3.1 [23] with power = .8, effect size = .2, predictors = 5, revealed that the minimum sample size required was 145. Participants were recruited via a call for participants published in the two editions of the CoronaCheck newsletter released on the 11^th^ and 18^th^ of December 2020.

In total, 1576 Australian participants took part in our study by completing our online survey. The data from 29 participants were excluded due to being duplicate responses, and 150 further responses were excluded due to having >20% missing data. The final sample therefore consisted of 1397 participants. Demographics for the final sample are provided in Table 1. Participants were also asked to provide details regarding their engagement with news and the CoronaCheck newsletter. This is summarised in Table 2.

**Table 1:** Sample demographics.

Demographic Variable	n	%
*Gender*		
Female	725	51.9
Male	648	46.4
Other	1	0.07
Prefer not to say	6	0.4
Did not answer	17	1.2
*Age*		
18–29	17	1.2
30–39	41	2.9
40–49	98	7
50–59	257	18.4
60–69	572	40.9
70–79	368	26.3
80+	42	3
Did not answer	2	0.1
*Highest Educational Level Completed*		
Did not complete school	60	4.3
Year 12 or equivalent	241	17.3
Diploma	270	19.3
Bachelor’s Degree	420	30.1
Postgraduate Degree	404	28.9
*Work Industry*		
Retired	428	30.6
Education and Training	198	14.2
Health care/social assistance	153	11
Professional, scientific and technical services	91	6.5
Information media and telecommunications	65	4.7
Administration and support	49	3.5
Public administration and safety	48	3.4
Arts and recreation	46	3.3
Financial/insurance services	34	2.4
Transport, postal and warehousing	20	1.4
Retail trade	19	1.4
Construction	19	1.4
Did not answer	227	16.2

**Table 2:** Engagement with news and CoronaCheck newsletter.

Variable	n	%
*Main news platform*		
Television	234	16.8
News aggregators	347	24.8
Social media feed	122	8.7
Messaging service	18	1.3
Online newspapers	423	30.3
Radio	159	11.3
Other	11	0.8
Did not answer	83	6
*How did you hear about the newsletter*?		
RMIT ABC Fact Check Website	824	59
ABC (including blogs, podcasts, website, tv)	112	8
Social Media	256	18.3
RMIT (email, website)	4	0.4
Friend/family member/employer	69	4.9
Misc Media	132	9.4
*Motivation for subscribing to CoronaCheck*		
Seeking accurate COVID-19 information, concern about misinformation	699	50
Debunk false information	236	16.9
General interest in COVID-19 information/misinformation	247	17.7
Check facts found online	97	6.9
Other	52	3.7
Did not answer	66	4.7
*Frequency of reading newsletter*		
Never	10	0.7
Only first time received	6	0.4
Rarely	38	2.7
Access each time but read sometimes	332	23.8
Every time received	1009	72.2
Did not answer	2	0.02

### 2.2 Procedure

Ethics approval was obtained from the Human Research Ethics Committee, RMIT University (approval number: 2020-23847-13060). Consent was implied by submission of the completed anonymous survey. Written consent was not appropriate given the anonymous nature of the survey. In the unlikely event of distress as a result of participation in the study, contact details for appropriate support services were provided in the participant information statement.

Participants completed the anonymous the survey online via Qualtrics which involved a series of measures outlined below.

### 2.3 Measures

**Demographics**. Non-identifying demographic information was provided by participants including age, gender, highest educational level, and work industry.

**Engagement with CoronaCheck newsletter**. Participants indicated the following: (i) where they first heard about the newsletter; (ii) their reason for subscribing; (iii) their frequency of reading the newsletter; (iv) the number of times they had shared debunked information from the newsletter in the last 3 months (response options were <5, 5–10, 10–20 and >20); and, (v) whether information in the newsletter has changed their mind (response options were Yes, Maybe and No).

**Engagement with news**. Participants indicated the following: (i) main news platform; (ii) frequency of sharing or re-posting claims; (iii) level of concern about fake news (response options ranged from 1–10, with 1 = not at all concerned and 10 = extremely concerned); (iv) capacity to distinguish fake news from genuine news (response options were very capable, moderately capable, a little bit capable, not at all capable and unsure); (v) whether they have shared claims that they later discovered were false (response options were Yes and No); and, (vi) whether they have shared possible misinformation, i.e. information for which the truth value was not known to them at the time of sharing (response options were Yes and No).

**Attitudes to COVID-19**. Participants indicated the following: (i) the extent to which they perceive expert information about COVID-19 to be trustworthy (response options ranged from 1–10, with 1 = not at all, and 10 = extremely); (ii) the extent to which they perceive COVID-19 vaccine-related information to be trustworthy (response options ranged from 1–10, with 1 = not at all, and 10 = extremely); (iii) how likely they are to have a COVID-19 vaccine when it is available (response options ranged from 1–10, with 1 = not at all, and 10 = extremely); and, (iv) factors that impact on their decision to have the vaccine (response options included vaccine efficacy, side effects, number of people who have had the vaccine, recommendation by health authorities and pre-existing health issues).

**Conspiracy mentality**. Susceptibility to conspiracy ideation was measured using the Conspiracy Mentality Questionnaire (α = .83; [24]). This is a 5-item scale which employs an 11-point rating scale ranging from 1 = Disagree very strongly to 11 = Agree very strongly. A sample item is “Many very important things happen in the world that the public is not informed about”. In the present study, internal consistency was high (Cronbach’s alpha = .80).

**Belief in science**. Belief in science was measured using the Credibility of Science Scale, α = .94 [25]. This is a 6-item scale which employs a 7-point rating scale ranging from 1 = Disagree very strongly to 7 = Agree very strongly. A sample item is “A lot of scientific theories are dead wrong”. All items were reverse scored so that higher scores reflected a greater belief in science. In the present study internal consistency was very high (Cronbach’s alpha = .89).

## 3. Results

Analyses were undertaken using SPSS (Version 26). Tables 3 and 4 present descriptive statistics for the study variables. On average, our sample had a higher belief in the credibility of science compared with other samples. For instance, in Lobato, Powell [18], the mean belief in the credibility of science was 3.40, (SD = 1.67), which was significantly lower than our sample, t(485.88) = 27.75, p = .0001, Mean Difference = 2.42, 95% CI [2.25, 2.59]. Conspiracy mentality was significantly lower in the current sample than Lobato, Powell [18], M = 7.99, SD = 1.80, t(1760) = -21.29, p = .0001, Mean Difference = -2.09, 95% CI [-2.29, -1.90].

**Table 3:** Frequency data for dichotomous study variables.

Variable	n	%
*How frequently have you shared debunked information in the past 3 months*?		
<5	976	69.9
5–10	305	21.8
10–20	75	5.4
>20	32	2.3
Did not answer	9	0.6
*Has information in the newsletter changed your mind*?		
Yes	129	9.2
Maybe	521	37.3
No	736	52.7
Did not answer	11	0.7
*Have you shared possible misinformation*?		
Yes	339	24.3
No	1052	75.4
Did not answer	6	0.4
*Reason for sharing possible misinformation (Totals are out of 339)*		
Seemed interesting	121	35.7
To get a second opinion about claim accuracy	130	38.3
For entertainment	42	12.4
To support a cause	24	7.1
Warn others	8	2.4
Did not answer	14	4.1
*Have you shared information that you later discovered was false*?		
Yes	440	31.4
No	942	67.4
Did not answer	15	1.1

**Table 4:** Summary data for study variables.

Variable	M	SD	Range	Skewness	Kurtosis
Frequency of sharing or reposting news items	3.26	2.52	1–10	.45	-.70
Frequency of reading posts in social media that were later debunked in CoronaCheck	4.58	2.96	1–10	-.16	-1.06
Level of concern about fake news	8.65	1.7	1–10	-1.78	3.49
Capacity to differentiate fake news from genuine news	4.23	.70	1–5	-1.16	3.28
Perceived trustworthiness of expert COVID-19 information	8.40	1.38	1–10	-1.48	3.95
Perceived trustworthiness of COVID-19 vaccine information	8.32	1.54	1–10	-1.56	3.76
Likelihood to have vaccine	8.61	1.98	1–10	-2.02	4.36
Conspiracy mentality	5.90	1.71	1–11	1.04	.37
Belief in science	5.82	.94	1–7	-1.13	1.81

Frequency data for dichotomous study variables is presented in Table 3. As can be seen in the table, approximately 30% had shared debunked information five times or more in the past 3 months, 24% had shared possible information and 31% had shared information later discovered to be false.

Summary data for (non-dichotomous) study variables is presented in Table 4. Overall, the current sample have a high concern about fake news, believe themselves to be good at discriminating fake news from genuine news, and trust expert COVID-19 information.

Correlations between study variables are presented in Table 5. Given that a number of the variables are dichotomous, Point-Biserial correlations were used.

**Table 5:** Point-Biserial correlations between study variables.

		1	2	3	4	5	6	7	8	9	10	11
1.	Age											
2.	Gender	.13**										
3.	Highest Educational level completed	.07**	-.11									
4.	Frequency read information later debunked in CoronaCheck	-.02	-.03	-.04								
5.	Information in newsletter changed mind	.06*	.03	.01	-13**							
6.	Capacity to differentiate fake news from genuine news	-.01	.06*	-.01	.05	.12**						
7.	Shared information not sure true	-.01	.11*	.03	.05	-13**	-11**					
8.	Perceived trustworthiness of expert COVID-19 information	.02	-.02	-.01	.00	.08**	.13**	-.05*				
9.	Perceived trustworthiness of vaccine information	.04	.02	.01	.03	.10**	.10**	-.04	.74**			
10.	Likelihood to have the COVID-19 vaccine	.01	.08**	.03	.03	.02	.09**	.01	.39**	.62**		
11.	Conspiracy mentality	.15**	-.01	-.18*	.06*	-12**	-12**	.06*	-26**	-30**	-24**	
12.	Belief in science	-13**	-.03	.15**	-.02	.10**	.15**	-09**	.43**	.44**	.38**	-42**

* = p < .05,

** = p < .01.

### 3.1 Predictors of likelihood to have COVID-19 vaccine

A multiple regression analysis was undertaken with likelihood to have the COVID-19 vaccine as the criterion variable and age, gender, highest educational level, work industry, conspiracy mentality, and belief in science as the predictors. Using the Enter Method, it was found that the overall model was significant, F(4, 1185) = 54.70, p = .0001, Adjusted R^2^ = .15. Beta, B, t and p values for predictors are presented in Table 6. Gender, belief in the credibility of science and conspiracy mentality emerged as significant predictors. Note that the gender variable was coded as follows: Female = 1, Male = 2, Other = 3, Prefer not to say = 4. (In total, only 1.6% of the sample indicated Prefer not to say or Other or did not answer the question and therefore only participants who selected male or female were included in analyses involving gender.).

**Table 6:** Beta, B, t, and p values for predictors for the multiple regression analysis.

	*Beta*	*ß*	*t*	*p*
Age	.04	.06, 95% CI [-.03,.17]	1.29	.19
Gender	.08	.31, 95% CI [.10,.52]	2.89	.004
Educational Level	-.01	-.03, 95% CI [-.12,.06]	-.72	.47
Work Industry		-.04, 95% CI [-.09,.01]	-1.61	.11
Conspiracy mentality	-.10	-.20 95% CI [-.31, -.08]	-3.38	.0001
Belief in science	.34	.71, 95% CI [.59,.83]	11.38	.0001

### 3.2 Predictors of sharing possible misinformation

A binary logistic regression analysis (see Table 7) was undertaken with sharing of possible misinformation as the criterion variable and age, gender, highest educational level, work industry, conspiracy mentality, and belief in science as predictors. Gender, highest educational level and belief in science emerged as significant predictors.

**Table 7:** Logistic regression analysis.

*Predictor*	ß	SE ß	Walds *x*^2^	*df*	*p*	*e* ß (odds ratio)
Age	-.08	.06	1.64	1	.201	.928
Gender	-.52	.13	15.17	1	.0001	.594
Educational Level	.12	.06	4.12	1	.04	1.13
Work Industry	.13	.16	.66	1	.42	1.14
Conspiracy mentality	.11	.07	2.42	1	.119	1.12
Belief in science	-.19	.07	6.29	1	.012	.831
Tests			*x* ^2^	*df*	*p*	
*Overall model evaluation*						
Likelihood ratio test			34.51	6	.0001	
Score test			34.53	6	.0001	
Wald test			287.08	1	.0001	
*Goodness of fit test*						
Hosmer & Lemeshow test			2.69	8	.95	

### 3.3 Willingness to share unverified content

We also determined the characteristics of participants who had shared possible misinformation. Of the 1397 participants, 339 (24%) indicated that they had shared unverified content. A series of independent samples t-tests were undertaken to compare the group who shared such content and the group who did not. A significantly higher belief in science was found in the group who did not share possible misinformation (M = 35.22, SD = 5.57) compared to those who did (M = 34.08, SD = 5.92), t(1303) = 3.14, p = .002, Mean difference = 1.14, 95% CI [.43, 1.85]. A significantly lower conspiracy mentality was found in the group who did not share possible misinformation (M = 19.53, SD = 5.21) compared to those who did (M = 20.29, SD = 4.89), t(1352) = -2.31, p = .021, Mean difference = -.75, 95% CI [-1.39, -.11].

## 4. Discussion

The CoronaCheck newsletter was developed to inoculate against the plethora of misinformation brought along with the COVID-19 pandemic. Here, we investigated whether subscribers to CoronaCheck were willing to share possible misinformation and, if so, whether the predictors of possible misinformation sharing were comparable to predictors in a general sample. We also investigated predictors of COVID-19 vaccination acceptance. Given that at least 70% of the population must be vaccinated to achieve herd immunity, it is important to investigate factors that undermine vaccine acceptance.

Our results revealed that even among a population who are concerned about misinformation and actively seek the debunking of misinformation, there is still a substantial inclination to share possible misinformation. This is surprising because one would predict that such individuals would be aware of the dangers associated with sharing misinformation and hence would exercise more caution. In this study, we included a very specific sample and thus findings may not be generalisable to the general population.

The majority of the extant literature located focusses on the susceptibility to *believe* misinformation rather than the propensity to *share* misinformation; and therefore only literature that explicitly addresses misinformation-sharing will be discussed here.

### 4.1 Concern about misinformation and capacity to judge claim veracity

On average, this sample was very concerned about misinformation and perceived themselves to have a very good ability to distinguish fake news from genuine news. This is unsurprising given the cohort included in the study, viz. subscribers of a fact checking newsletter. However, what is interesting is that 24% of this cohort were willing to share possible misinformation and 31% had shared information that they later discovered to be false. Participants were asked why they shared information of whose truth value they were uncertain. Approximately 37% indicated that it seemed interesting, 38.3% shared to get a second opinion about the claim’s veracity and 12.4% shared information for its entertainment value.

Given that sharing of possible misinformation has significant potential to engender false beliefs in others, this practice is problematic even when done without ill intent. Once information is shared and re-shared, the original sharer’s intention is lost and therefore what may begin as “a joke is transformed into a lie” [16]

### 4.2 Predictors of possible misinformation sharing

Males were more likely to share possible misinformation (that is, information whose truth value is unknown to the sharer at the time of sharing) than females. Sharing of possible misinformation was positively predicted by educational level and negatively predicted by belief in the credibility of science. Interestingly, Lobato et al. [18] did not find any variation in misinformation sharing as a function of belief in the credibility of science. Rather they noted that aspects of political belief particularly social dominance orientation, explained the inclination to share misinformation [18]. While we found no age effects (possibly due to the limited age variation in our sample), Guess et al. [17] found that individuals aged 65 years and over and politically conservative individuals were more likely to share fake news and misinformation.

In the present study, we did not assess political orientation as in the Australian context, there is less political polarization and it seems to play less of a role in determining behavior than in countries such as the US. However, it is of interest that in the study by Lobato et al. [18] belief in science made no contribution to the inclination to share misinformation demonstrating that in our sample, different predictors played in a role in misinformation sharing.

Guess et al. [17] found that although Facebook users shared links frequently during the 2016 US presidential election, the sharing of information from fake news sites was only done by 10% of their sample of 3500 participants, with higher numbers of Republicans sharing such information than Democrats. Although it is not possible to directly compare this finding with ours as we did not explicitly assess whether participants shared information from fake news sites, it is nevertheless worth noting that, in our sample, 24% shared possible misinformation and 31% had shared information that they later discovered to be false. This seems higher than one would expect simply due to bad luck. Rather it seems that this is more likely to occur due to a limited ability to distinguish fake news from genuine news and a lack of caution with information sharing.

### 4.3 Predictors of COVID-19 vaccination acceptance

Although the sample tended to report a high level of COVID-19 vaccine acceptance (approximately 82%), this varied as a function of individual differences. Vaccine acceptance was positively predicted by belief in science and negatively predicted by conspiracy mentality. Moreover, females typically indicated a higher vaccine acceptance than males. Interestingly, in contrast with our findings, others have found that females report more vaccine hesitancy than males [19,26]. This is also reflected in a recent survey by Essential Research of 1,090 participants from the general Australian public, where 20% of females and 12% of males indicated that they would “never get vaccinated” [21].

### 4.4 Limitations and suggestions for further research

The sample included in the present study represents, by design, a population who are especially concerned about fake news and misinformation. Thus, our findings are not generalisable to the general population. It may be even the case that our findings are not generalisable to CoronaCheck newsletter subscribers in general as there may be relevant differences (for instance, concerning information sharing) between subscribers who participated in the study and those who chose not to participate. We also note that our sample was highly educated (nearly 60% had completed at least a Bachelors Degree) and 30.6% were retired. Although political orientation is a less salient predictor of behavior in the Australian context, than in countries such as the US, it would be of value in future research to determine political orientation even when including an Australian sample.

When measuring engagement with news and attitudes to COVID-19 and its associated vaccine, we used single item measures. Although these are efficient, we acknowledge that there are limitations associated with such measures.

In future research, it would be of value to present this cohort with both genuine and fake news claims in order to determine whether the high self-reported capacity in our cohort to distinguish fake news from genuine news is borne out.

Although it is possible that reading the CoronaCheck newsletter would put accuracy at the forefront of attention when encountering news claims, this may not be a sufficiently explicit ‘intervention’ to reduce the sharing of misinformation. In future research it would therefore be of interest to investigate whether interventions such as making accuracy more salient [12] would discourage sharing of possible misinformation in this cohort.

## 5. Conclusion

In this research, we studied the attitudes and behaviour of a sample of subscribers to the CoronaCheck newsletter, a publication focussed on the debunking of misinformation about COVID-19. Participants reported a high awareness of misinformation and rated themselves as highly capable of differentiating misinformation from accurate information. Surprisingly, despite this, a substantial proportion of respondents reported that they have in the past shared information the accuracy of which they were unsure or which they knew to be inaccurate.

It is puzzling that sharing of possible misinformation persists in a cohort who are both attuned to and concerned about misinformation and who actively seek the debunking of misinformation. Sharing misinformation, even when it is not done to deceive, increases the chances that it will promote faulty beliefs in others. The spread of misinformation undermines efforts to control COVID-19 by reducing compliance with measures, including vaccination, to curtail its spread. While subscribing to a fact checking newsletter such as CoronaCheck likely sensitises individuals to the importance of information accuracy, this is clearly not sufficient to prevent individuals from sharing possible misinformation. It therefore appears that, even for this cohort, a more explicit intervention is required to deter misinformation sharing.
